# Phospholipid transfer protein ameliorates sepsis-induced cardiac dysfunction through NLRP3 inflammasome inhibition

**DOI:** 10.1515/med-2024-0915

**Published:** 2024-03-27

**Authors:** Jian Wang, Jing Hou, Chaohua Peng

**Affiliations:** Emergency and Intensive Care Medicine Center, Guang’an People’s Hospital, Guang’an city, Sichuan 638500, PR China

**Keywords:** SICD, PLTP, NLRP3 inflammasome, GSDMD, pyroptosis

## Abstract

Cardiomyocyte pyroptosis is a primary contributor to sepsis-induced cardiac dysfunction (SICD). Recombinant phospholipid transfer protein (PLTP) have been demonstrated to possess anti-inflammatory and antiseptic properties. However, the effect of PLTP on SICD remains unknown. In this study, we established the *in vivo* and *in vitro* sepsis model with the recombinant PLTP treatment. The survival rates of mice, mouse cardiac function, cell viability, the protein level of proinflammatory cytokine, and lactate dehydrogenase level were evaluated. The cardiomyocyte pyroptotic changes were observed. The distribution of PLTP and NOD-like receptor thermal protein domain associated protein 3 (NLRP3) in mouse myocardial tissue and expression of PLTP, apoptosis associated speck like protein containing a CARD (ASC), NLRP3, caspase-1, interleukin (IL)-1β, and Gasdermin D (GSDMD) were detected. PLTP ameliorated the cecal ligation and puncture-induced mouse survival rate decrease and cardiac dysfunction, inhibited the IL-1β, IL-18, and tumor necrosis factor (TNF)-α release, and blocked the NLRP3 inflammasome/GSDMD signaling pathway in septic mice. *In vitro*, PLTP reversed the lipopolysaccharide-induced cardiomyocyte pyroptosis, expression of IL-1β, IL-6, TNF-α, and activation of the NLRP3 inflammasome/GSDMD signal pathway. Moreover, PLTP could bind to NLRP3 and negatively regulate the activity of the NLRP3 inflammasome/GSDMD signal pathway. This study demonstrated that PLTP can ameliorate SICD by inhibiting inflammatory responses and cardiomyocyte pyroptosis by blocking the activation of the NLRP3 inflammasome/GSDMD signaling pathway.

## 1 Introduction

Sepsis, which has a high mortality rate of almost 30% [1], is a severe systemic inflammatory response caused by bacterial infection that can cause multiple-organ dysfunction [2]. Cardiac dysfunction is a serious complication of sepsis that was observed in almost 40% of such patients [3]. Cardiac dysfunction increases the mortality rate of patients with sepsis by up to 50%, compared to 20% in patients with sepsis without cardiac dysfunction [3]. In recent years, supportive therapy and intensive care technology in the treatment of sepsis have made great progress; however, the mortality rate of sepsis-induced cardiac dysfunction (SICD) remains high [4]. Therefore, it is of great significance to elucidate the complex pathogenesis of SICD and explore new therapeutic strategies.

Cardiomyocytes are critical for cardiac function. However, they are nonrenewable cells, their death plays a key role in cardiac dysfunction [5]. The inflammatory response affects cardiac function and the death mode of cardiomyocytes in sepsis [6]. In sepsis, bacterial infection induces the excessive release of inflammatory cytokines, such as tumor necrosis factor (TNF)-α, interleukin (IL)-1, and IL-18, causing cellular injury and multiple-organ dysfunction syndrome [7,8]. Lipopolysaccharide (LPS) is the main component of the cell wall of Gram-negative bacteria, which is an important substance in the inflammatory response of sepsis [9]. It has been reported that cardiomyocytes has the ability to secrete IL-1β, IL-18, and TNF-a after LPS treatment [10–12]. Moreover, sepsis-induced cardiac inflammation has been reported to be involved in pyroptosis [13]. It is well known that inflammatory cytokines (TNF-α, IL-1β, and IL-18) are involved in the occurrence of pyroptosis [14]. Pyroptosis of cardiomyocytes within the myocardium has been observed in patients with sepsis [15]; however, its pathogenesis is not yet fully understood.

Phospholipid transfer protein (PLTP) is a hydrophobic glycoprotein that is widely expressed in eukaryotes, and it transfers amphiphilic lipids between circulating lipoproteins and among lipoproteins, cells, and tissues [16]. Moreover, PLTP plays an important role in the regulation of inflammation [17]. It was reported that PLTP can alleviate LPS-mediated inflammation and sepsis [16]. Recombinant human plasma PLTP (rh PLTP) has been used to prevent bacterial growth and treat sepsis [18]. However, the effects of PLTP on SICD have not yet been reported, and the mechanism is unclear.

Pyroptosis is a form of lytic programmed cell death initiated by inflammasomes [19]. The caspase-dependent pyroptosis is characterized by the activation of pathways leading to the activation of NOD-like receptors, especially the NLRP3 inflammasome, which is an oligomeric complex containing NLRP3, ASC, and Caspase-1 [20]. The activated NLRP3 inflammasome drives activation of caspase-1, which cleave Gasdermin D (GSDMD), and ultimately the release of IL-1β through cell membrane rupture [19]. Pyroptosis plays an important role in the occurrence and development of cardiovascular diseases, especially in patients suffering from myocardial infarction [21], hypertension [22], and cardiomyopathy [23], as well as in animal models of ischaemia–reperfusion injury [24]. It has been reported that pyroptosis is involved in development of SCID [15]; however, the mechanism remains unclear.

In the present study, cecal ligation and puncture (CLP) was used to generate a mouse model of sepsis, and mouse cardiomyocyte (M6200 cells) were treated with LPS to mimic sepsis-induced inflammation in cardiomyocytes. Both models were treated with rh PLTP to explore its effects on SICD and its potential mechanism.

## 2 Materials and methods

### 2.1 CLP and sham operation

After anesthesia was induced with 2% isoflurane, the cecum of each mouse was completely exposed via an abdominal surface incision. Then, 70% of the total length of the cecum was ligated with 4–0 silk, and a penetrating puncture was performed with a No. 22 needle (BD Biosciences, USA). Sham-operated mice underwent the same operation as CLP mice but without ligation and puncture of the cecum.

### 2.2 Animals and treatment

The C57BJ/6 mice (male, 8 weeks old, body weight = 23 ± 2 g) were obtained from the Chongqing Medical University Animal Center (Chongqing, China). The mice were fed water or food and kept in a specific pathogen-free animal room. The mice were randomly divided into the Sham, rh PLTP, CLP, and CLP + rh PLTP groups (*n* = 5 per group). Sham group: mice in the Sham group underwent sham operation. rh PLTP group: mice in the rh PLTP group underwent sham operation; meanwhile, an intraperitoneal injection of rh PLTP was performed (25 µg of active PLTP in a volume of 200 µL of sterile water, Abmart Pharmaceutical Technology Co., Ltd, Shanghai, China) [18]. CLP group: mice in the CLP group underwent CLP. CLP + rh PLTP group: mice in the CLP + rh PLTP group underwent CLP; meanwhile, an intraperitoneal injection of rh PLTP was performed. After 24 h of CLP, the mice were anesthetized with 2% isoflurane and killed by cervical dislocation, then the serum of mice were collected for ELISA test and the hearts of mice were collected for immunohistochemistry (IHC) and western blotting (WB).


**Ethics approval and consent to participate:** The animal experiment was allowed by the Animal Ethics Committee of Guang’an People’s Hospital (Animal Experimental Ethical Inspection Form of Guang’an People’s Hospital No. 2200214).

### 2.3 Survival studies

Survival rates were analyzed using the GraphPad software. In brief, 40 mice were randomly divided into the Sham, rh PLTP, CLP, and CLP + rh PLTP groups. The mice in the CLP + rh PLTP and rh PLTP groups received intraperitoneal injections of rh PLTP (25 µg of active PLTP in a volume of 200 µL of sterile water) once daily. Survival curves were plotted every 6–12 h for 7 consecutive days. After 7 days, 2% isoflurane was used to anesthetize the surviving mice, which were sacrificed via cervical dislocation. The data were analyzed using GraphPad software.

### 2.4 IL-1β, IL-18, and TNF-α detection

IL-1β, IL-18, and TNF-α levels in the serum of mice were measured using ELISA kits (Abmart). In brief, blood was collected from mice in the four groups, centrifuged at 1,000×*g* at 4°C for 10 min, and the supernatant was collected and analyzed using ELISA.

### 2.5 IHC

IHC was performed to evaluate the distribution of PLTP and NLRP3 in the heart tissues of mice. Paraffin sections of heart tissue were deparaffinized in xylene and rehydrated via successive incubations with 100, 90, 80, and 70% alcohol. Endogenous peroxidase was blocked using a hydrogen peroxide block solution. The sections were incubated at 4°C overnight with primary antibodies against PLTP (Abmart) and NLRP3 (Abmart). Then, HRP polymer was added dropwise, and the sections were incubated at room temperature for 30 min. DAB substrate was added dropwise for staining. Finally, the sections were rinsed with tap water, re-stained, dehydrated, cleared, and sealed. The images were taken (magnification, ×400) using an optical microscope (LEICA DFC550 DM4 B).

### 2.6 Echocardiography

Left ventricular function in mice was assessed using echocardiography (Philips TIS 0.8, Koninklijke Philips N.V.) and an RMV 707B transducer. The mice were anesthetized with 2% isoflurane before echocardiography. Left ventricular fractional shortening (LVFS, %) and left ventricular ejection fraction (LVEF, %) were automatically calculated by echocardiography.

### 2.7 Cell culture and treatment

The mouse cardiomyocyte M6200 cell line was purchased from Zhong Qiao Xin Zhou Biotechnology Co., Ltd (Shanghai, China). The cells were cultured in Dulbecco’s Modified Eagle’s Medium (Thermo Fisher Scientific) containing 10% fetal bovine serum (PAN-Biotech GmbH, Adenbach, Germany) in a humidified incubator (5% CO_2_ at 37°C). For LPS treatment, M6200 cells were cultured with or without 0.1, 1.0, 10, or 100 mg/L LPS (MedChemExpress Co., NJ, USA) for 24 h, followed by treatment with 125 mg/L rh PLTP (Abmart). Then, the cells were collected for subsequent experiments.

### 2.8 Cell counting kit‐8 (CCK-8) assay

The viability of M6200 cells was detected using CCK-8 (Beyotime, Shanghai, China) according to the manufacturer’s protocols. In brief, M6200 cells were seeded at a density of 5 × 10^3^/well in 96-well microplates. Cells were exposed to various concentrations of LPS (0, 0.1, 1.0, and 10 mg/L) for 24 h, and 10 µL of CCK-8 reagent was added to each well, followed by incubation for 1 h in a 37°C incubator. The absorption of each well was read at 450 nm on a microplate reader (Bio‐Rad, Hercules, CA, USA). The cell viability was calculated based on the absorbance.

### 2.9 Lactate dehydrogenase (LDH) release assay

An LDH assay kit (Solarbio Company, Shanghai, China) was used to detect LDH levels in the supernatant of M6200 cells. M6200 cells were treated with or without LPS (0.1, 1, or 10 mg/L) for 24 h. Then, the supernatant was collected and subjected to an LDH assay. Finally, treated samples were detected via a colorimetric assay at 450 nm, and LDH concentrations were calculated and analyzed.

### 2.10 Scanning electron microscopy (SEM)

Sterilized cover glass slides (1.2 × 1.2 cm^2^) were placed in a six-well plate and cultured with the cell suspension until cells reached the exponential proliferation phase. Subsequently, the slides were rinsed twice with PBS (pH 7.4), fixed with 25% glutaraldehyde for 30 min, and rinsed thrice with PBS. Then, 1% osmium tetroxide was added for 45 min for fixation, followed by three rinses with PBS. The samples were dehydrated by soaking them in an acetone/isoamyl acetate solution for 10 min and an isoamyl acetate solution for 30 min. Subsequently, the samples were removed, subjected to critical point drying and gold spraying, and finally photographed using an electron microscope (Hitachi HT7700, Tokyo, Japan). The photos were recorded at ×2,000 and ×7,000 magnifications.

### 2.11 Transient siRNA transfection

The siRNAs were purchased from Shanghai GeneChem Co., Ltd (Shanghai, China). The M6200 cells were transfected with siRNA-NLRP3 (10 nmol/L) and siRNA-NC (10 nmol/L) using the Lipofectamine™ 3000 regent (Thermo Fisher Scientific, USA), according to the manufacturer’s recommendation. After 48 h, cells were stimulated with 10 mg/L LPS or 125 mg/L rh PLTP, then, cellular protein were harvested for WB.

### 2.12 Co-immunoprecipitation (Co-IP)

Co-IP was performed to determine the interaction of PLTP with NLRP3 using an immunoprecipitation kit with protein A + G agarose gels (Beyotime Biotechnology). The procedure was performed as follows: a volume of 12.5 μL of primary antibody, 25 μL of protein A-G agarose gel suspension, and 250 μL of PBS was mixed and incubated together for 2 h at 4°C. Then, the cellular protein was added and incubated together with primary antibody–protein A-G agarose compound overnight at 4°C on a rocking platform. Primary antibodies targeting PLTP (2 μg, PL0301243, PL Laboratories Inc.), NLRP3 (2 μg, T55651, Abmart), and IgG (2 μg, BL003A, Bio-Sharp, Shanghai, China) were used. The next day, the cells were centrifuged at 3,000 rpm at 4°C and washed twice with PBS containing protein inhibitors, followed by WB with the primary antibodies used for the Co-IP experiments.

### 2.13 WB

The 30 μg quantified protein from mouse heart sample or M6200 cells were electrophoresed in a 10% SDS-PAGE gel and transferred to a PVDF membrane. The membrane was blocked with 5% skim milk for 2 h and incubated with primary antibodies against PLTP (1:1,000, PK72825; Abmart), ASC (1:1,000, TP73812; Abmart), NLRP3 (1:1,000, T55651; Abmart), caspase-1 (1:1,000, TP70406; Abmart), IL-1β (1:1,000, PA1586; Abmart), GSDMD (1:1,000, TA4012; Abmart), and GAPDH (1:10,000, ab181602; Abcam) overnight at 4°C. The membrane was washed three times with Tris-buffered saline containing 0.1% Tween 20 and incubated with HRP anti-rabbit IgG antibody (1:10,000, ab184787; Abcam) at 20°C for 2 h. The protein brands were visualized by chemiluminescence and analyzed using Quantity One software 6.0.

### 2.14 Statistical analysis

GraphPad Prism version 9.0 was used for all statistical analyses. The data were expressed as the mean ± SD. The survival analysis between groups was done using Log-rank (Mantel-Cox) method. The unpaired *t*-test with two-tailed was used for two groups comparison. The one-way analysis of variance (ANOVA) followed by Dunnett’s test was used for comparing treated group with control group in Figure 4a and b, while the ANOVA followed by Turkey’s test was used for other figures when doing pair-wise comparison. Statistical significance was indicated by *P*-value of <0.05.

## 3 Results

### 3.1 CLP decreased the survival of mice and induced inflammatory factor release and cardiac dysfunction

**Figure 1:**
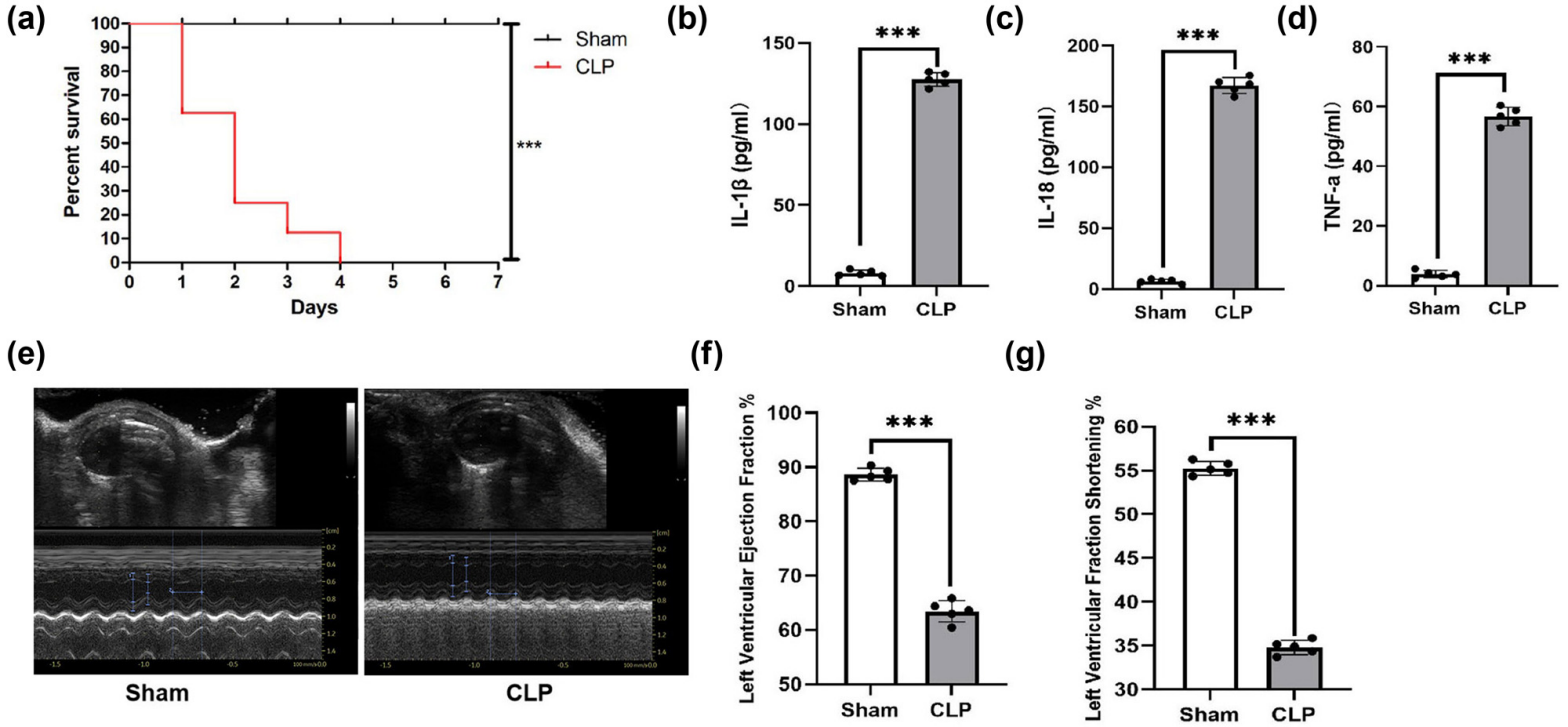
CLP decreased the survival of mice and induced inflammatory factor release and cardiac dysfunction. (a) Survival rates of mice in the CLP and Sham groups were compared. *n* = 8, *** = *P* < 0.001. (b)–(d) ELISA was performed to detect the serum levels of IL-1β, IL-18, and TNF-α in mice in the CLP and Sham groups. (e)–(g) Cardiac function of mice in the CLP and Sham groups was analyzed. *n* = 5, *** = *P* < 0.001.

CLP was performed to induce sepsis in mice. Then, the survival rate, inflammatory factor levels (IL-1β, IL-18, and TNF-α), and cardiac function were determined. All the mice in the CLP group died on Day 4, whereas the survival rate was 100% in the Sham group (Figure 1a). Serum IL-1β, IL-18, and TNF-α levels were significantly higher in the CLP group than in the Sham group (Figure 1b–d). In addition, LVEF and LVFS were significantly lower in the CLP group than in the Sham group (Figure 1e and f).

### 3.2 rh PLTP improved mouse survival, reduced the release of inflammatory factors, and alleviated cardiac dysfunction induced by CLP

**Figure 2:**
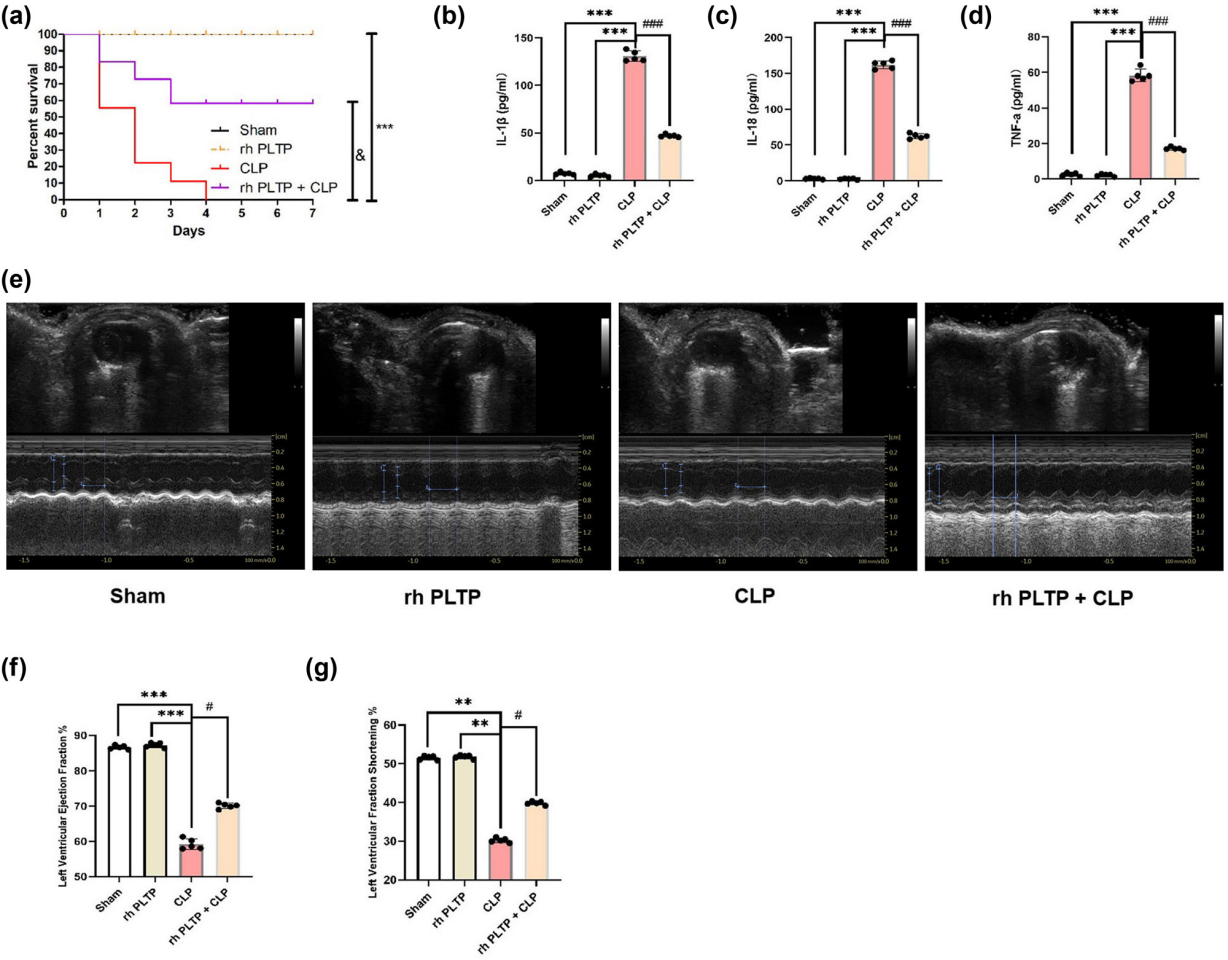
Effects of rh PLTP on survival rates, serum IL-1β, IL-18, and TNF-α levels, and cardiac function in mice with sepsis. (a) Survival rates of mice in the Sham, CLP, rh PLTP, and CLP + rh PLTP groups (*n* = 8/group) were analyzed. *** = *P* < 0.001, & = *P* < 0.01. (b)–(d) ELISA was performed to detect the serum levels of IL-1β, IL-18, and TNF-α in mice in the four groups. (e)–(g) Cardiac function of mice in the four groups was compared. *n* = 5, *** = *P* < 0.001, # = *P* < 0.05, ### = *P* < 0.001.

To determine the effect of PLTP on sepsis, the mice were intraperitoneally injected with rh PLTP, after which CLP was performed. The results showed that rh PLTP significantly ameliorated the CLP-induced decrease in mouse survival rates (Figure 2a). We also observed that rh PLTP reversed the CLP-induced release of IL-1β, IL-18, and TNF-α (Figure 2b–d) and cardiac dysfunction in mice (Figure 2e and f).

### 3.3 rh PLTP inhibited the activation of the NLRP3 inflammasome/GSDMD signaling pathway in the heart tissue of mice with sepsis

**Figure 3:**
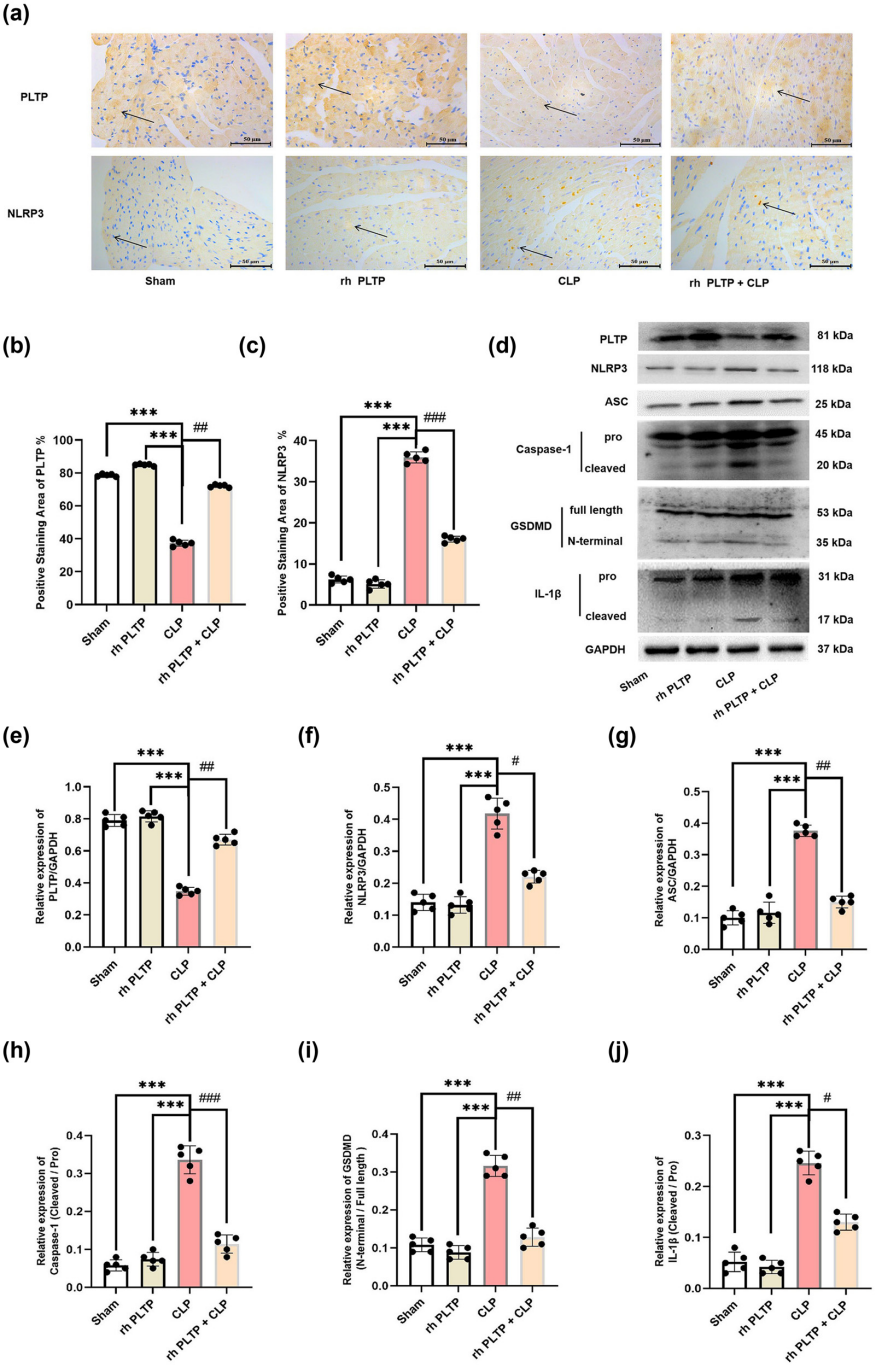
Effects of PLTP on the activation of the NLRP3 inflammasome/GS. DMD pathway in the heart tissue of mice with sepsis. (a)–(c) IHC was performed to evaluate the distribution of PLTP and NLRP3 in the heart tissue of mice, and the positive staining areas of PLTP and NLRP3 were analyzed. *n* = 5, *** = *P* < 0.001, ## = *P* < 0.01, ### = *P* < 0.001. (d)–(j) Expression of PLTP, NLRP3, ASC, caspase-1, IL-1β, and GSDMD was detected by WB, and the relative expression was calculated from the gray-scan value and analyzed using GraphPad Prism. The graph presents the densitometry results of three independent experiments (mean ± SD). *n* = 5, *** = *P* < 0.001, # = *P*. < 0.05, ## = *P* < 0.01, ### = *P* < 0.001.

Sepsis-induced cardiac inflammatory responses lead to pyroptosis [13], and our results showed that rh PLTP treatment inhibited inflammatory factor release in mice with sepsis (Figure 2b–d). Therefore, we examined whether PLTP regulates pyroptosis. IHC was performed to evaluate the distribution of PLTP and NLRP3 in the heart tissue of mice. The results demonstrated that PLTP was widely distributed in the cytoplasm and cell membrane, whereas NLRP3 exhibited dotted aggregation on the cell membrane. Moreover, rh PLTP treatment reversed the CLP-induced aggregation of NLRP3 in the heart tissue of mice (Figure 3a–c).

Activation of the NLRP3 inflammasome and GSDMD pathway is the key event in pyroptosis [25]. To further investigate the effect of PLTP on pyroptosis, the mice were intraperitoneally injected with rh PLTP, after which CLP was performed. In addition, PLTP, NLRP3, ASC, caspase-1, IL-1β, and GSDMD expression were determined using WB. The results showed that rh PLTP treatment reversed the CLP-induced decrease in PLTP expression and increase in NLRP3, ASC, caspase-1, IL-1β, and GSDMD expression (Figure 3d–j).

### 3.4 rh PLTP treatment inhibited LPS-induced pyroptosis in mouse cardiomyocytes

**Figure 4:**
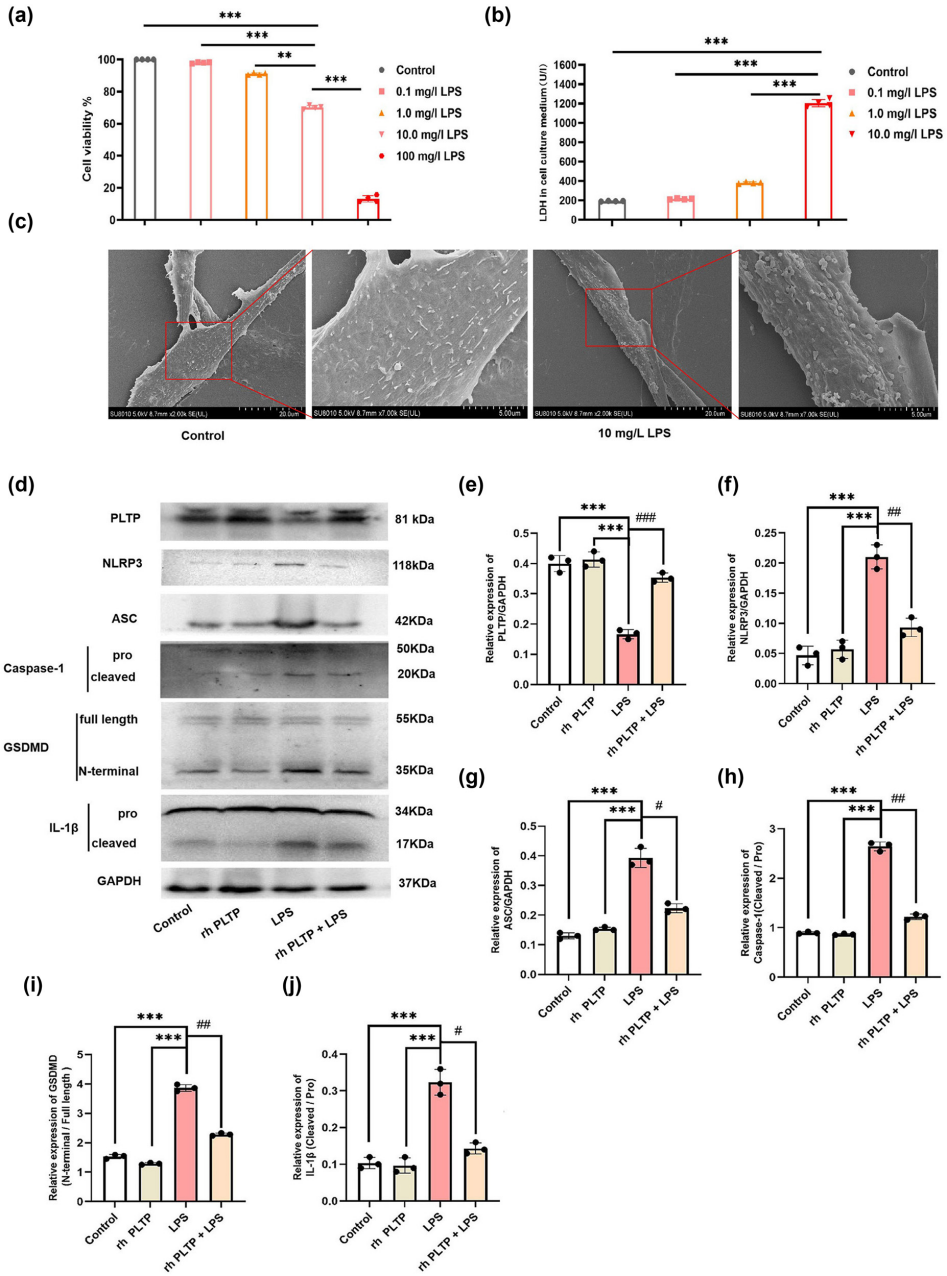
Effect of rh PLTP on LPS-induced pyroptosis in mouse cardiomyocytes. (a) Cell viability was detected by the CCK-8 assay. *n* = 4, ** = *P* < 0.01, *** = *P* < 0.001. (b) LDH content in the cell culture supernatant was detected by the LDH release assay. *n* = 4, *** = *P* < 0.001. (c) Morphological changes of the M6200 cell membrane were observed using SEM. (d)–(j) Expression of PLTP, NLRP3, ASC, caspase-1, IL-1β, and GSDMD was detected by WB, and the relative expression was calculated from the gray-scan value and analyzed using GraphPad Prism. *n* = 3, *** = *P* < 0.001, # = *P* < 0.05, ## = *P* < 0.01.

To investigate the effect of LPS on pyroptosis in mouse cardiomyocytes, we treated M6200 cells with LPS (0, 0.1, 1.0, 10, or 100 mg/L) for 24 h, and the CCK-8 assay was performed to detect cell viability. The results showed that LPS decreased cell viability in a concentration-dependent manner (Figure 4a). Then, M6200 cells were treated with LPS (0, 0.1, 1.0, or 10 mg/L) for 24 h, and the LDH content in the supernatant was measured. LPS-induced LDH release in a concentration-dependent manner (Figure 4b). We also observed the morphological changes of M6200 cells using scanning electron microscope. LPS treatment resulted in the formation of swelling vesicles on the surface of the cell membrane, also termed pyroptotic bodies (Figure 4c).

To investigate the effect of rh PLTP on LPS-induced pyroptosis in mouse cardiomyocytes, M6200 cells were treated with 10 mg/L LPS, followed by treatment with 125 mg/L rh PLTP for 24 h. We detected the expression of PLTP, NLRP3, ASC, caspase-1, IL-1β, and GSDMD via WB. The results showed that rh PLTP treatment reversed the LPS-induced decrease in PLTP expression, reversed the LPS-induced expression of NLRP3 and ASC, and inhibited the LPS-induced cleavage of caspase-1, IL-1β, and GSDMD (Figure 4d–j).

### 3.5 PLTP was bound to NLRP3 to mediate NRLP3 inflammasome/GSDMD pathway

**Figure 5:**
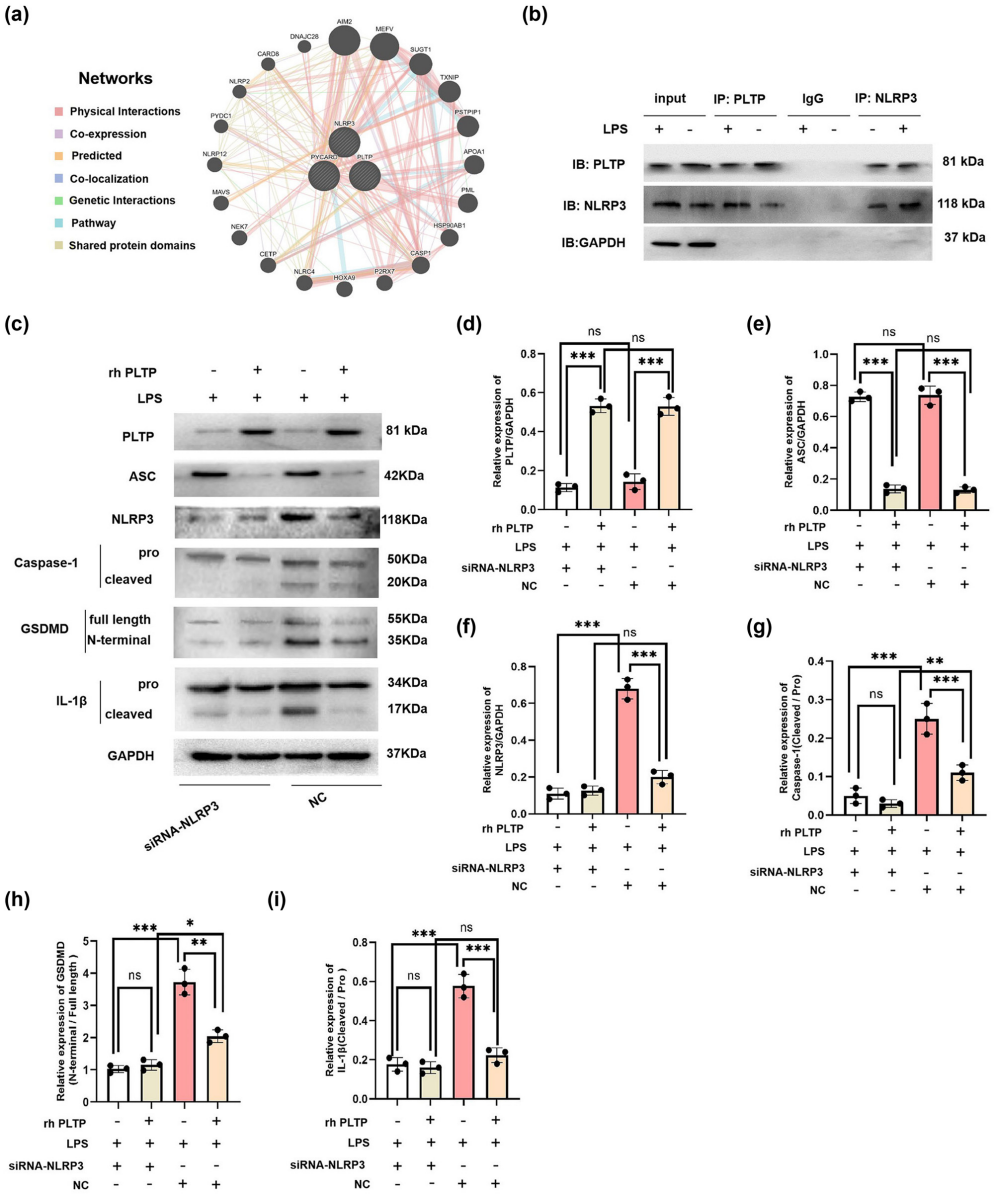
Interaction between PLTP and NLRP3 proteins. (a) Interaction between PLTP and NLRP3 was predicted using the GeneMANIA database. (b) Predicted relationship between PLTP and NLRP3 was verified by Co-IP, *n* = 3. (c)–(i) Expression of PLTP, NLRP3, ASC, caspase-1, IL-1β, and GSDMD was detected by WB, and the relative expression was calculated from the gray-scan value and analyzed using GraphPad Prism. *n* = 3, ns = *P* > 0.05, * = *P* < 0.05, ** = *P* < 0.01, *** = *P* < 0.001.

Our results demonstrated that PLTP inhibited CLP-induced activation of the NLRP3 inflammasome/GSDMD signaling pathway (Figure 3c–j) and LPS-induced cleavage of caspase-1, IL-1β, and GSDMD (Figure 4d–j). To clarify the mechanism, we assessed the interactions between PLTP and NLRP3 using the GeneMANIA database and the reference data from *Homo sapiens*, which indicated an interaction between PLTP and NLRP3 (Figure 5a). Subsequently, Co-IP was performed to verify the interaction between PLTP and NLRP3. The result showed that PLTP was bound to NLRP3 (Figure 5b).

To verify the regulatory relationship between PLTP and NLRP3 inflammasome/GSDMD pathway, we silenced the expression of NLRP3 using siRNA-NLRP3 and subsequently treated with LPS and rh PLTP. We found that rh PLTP treatment inhibited the LPS-induced activation of NLRP3 inflammasome/GSDMD signaling pathway, while silencing NLRP3 expression had no effect on the PLTP expression (Figure 5c–i).

## 4 Discussion

*In vivo*, we found that rh PLTP ameliorated decreases in survival, increases in IL-1β, IL-18, and TNF-α release, and cardiac dysfunction induced by CLP in mice. Moreover, we also found that rh PLTP treatment inhibited the activation of the NLRP3 inflammasome/GSDMD signaling pathway in the heart tissue of mice with sepsis. *In vitro*, we observed that rh PLTP treatment inhibited LPS-induced pyroptosis in mouse cardiomyocytes. In the mechanistic study, we demonstrated that PLTP binds to NLRP3 to inhibit the activation of the NLRP3 inflammasome/GSDMD signaling pathway, which is one of the main pathways mediating pyroptosis.

Sepsis is an overwhelming reaction to infection that leads to high morbidity and mortality [26]. According to reports, the hospital mortality rate was 17% for normal sepsis and up to 26% for severe sepsis. According to high-income country data, there are an estimated 31.5 million cases of sepsis and 19.4 million cases of severe sepsis per year, potentially resulting in 5.3 million deaths [27]. The severe inflammatory response caused by sepsis is responsible for the high mortality rate [28]. The pathophysiology of sepsis is characterized by hyperactive and dysregulated endogenous inflammatory mediators, including IL-1β, IL-18, and TNF-α [29,30]. Sepsis can lead to inflammatory damage in nearly every organ system [31]. The cardiovascular system is an important organ system that is frequently compromised by sepsis [4]. Cardiac dysfunction is one of the major complications of sepsis, and SICD is considered a leading cause of death in sepsis [32]. In our study, we observed in mice that CLP-induced sepsis resulted in decreased survival (Figure 1a), increased release of the pro-inflammatory cytokines IL-1β, IL-18, and TNF-α (Figure 1b–d), and cardiac dysfunction (Figure 1e–g).

Sepsis is mostly caused by endotoxin, which is released from Gram-negative bacteria [33]. LPS is an important component of endotoxin that can cause a cascade of immune stimulation and toxic pathophysiological activities in the body, thereby inducing cardiac dysfunction [34,35]. LPS is often used to generate models of inflammation-related diseases such as acute lung injury [36], neuroinflammation [37], and SICD [2]. Pyroptosis is a pro-inflammatory type of programmed cell death [38]. Accumulating evidence indicates that LPS can induce pyroptosis [39]. It has been reported that LPS can induce pyroptosis in hepatocytes [40], human bronchial epithelial cells [41], macrophages [42], and cardiomyocytes [15]. Our research found that LPS decreased cell viability and induced pyroptosis in cardiomyocytes (Figure 4a–c).

It was reported that LPS and CLP can activate the NLRP3 inflammasome [43]. The NLRP3 inflammasome consists of NLRP3, ASC, and procaspase-1 [44]. Upon activation, NLRP3 protein recruits the adapter protein ASC and subsequently induces the cleavage and activation of procaspase-1 [44]. Activated caspase-1 can cleave GSDMD to release GSDMD-N, which binds to phosphoinositol in the plasma membrane and generates a membrane pore with a diameter of approximately 12–14 nm. This leads to cell expansion and eventual rupture, releasing activated IL-1β and subsequently inducing pyroptosis [45]. In our study, we found that CLP or LPS treatment activated the NLRP3 inflammasome, leading to the accumulation of GSDMD-N and mature IL-1β. These findings demonstrated that CLP and LPS induced cardiomyocyte pyroptosis through NLRP3 inflammasome/GSDMD pathway activation (Figures 3 and 4d–j).

PLTP is widely expressed in eukaryotes [12]. The structure of the mouse PLTP gene is almost identical to that of the human gene, and the encoded amino acid sequence has 83% homology with the human PLTP protein [46]. PLTP is mainly involved in lipid metabolism [47], phosphatidylcholine synthesis [48], and the secretion and transmission of neurotransmitters [49]. Moreover, PLTP plays a key role in regulating immune responses, and it is directly linked to a wide range of inflammatory diseases, including bacterial infection-induced sepsis [16], which might be dependent on its ability to accelerate the reverse LPS transport pathway [50]. Our study found that rh PLTP treatment ameliorated the CLP-induced decrease in mouse survival, increase in inflammation factor release, and cardiac dysfunction (Figure 2).

Recently, the role and mechanism of PLTP in regulating cell death have attracted substantial attention. It was found that PLTP is involved in the regulation of cell apoptosis [51] and ferroptosis [52]. However, the regulatory relationship between PLTP and pyroptosis has never been reported. This study provides the first evidence that rh PLTP treatment can inhibit the CLP- or LPS-induced activation of the NLRP3 inflammasome/GSDMD pathway *in vivo* (Figure 3c–j) and *in vitro* (Figure 4d–j). Moreover, we found that PLTP could bind to NLRP3 to mediate NRLP3 inflammasome/GSDMD pathway, which is involved in the occurrence of pyroptosis (Figure 5).

In summary, our research indicated that PLTP bound to NLRP3 to impede the CLP- or LPS-induced activation of the NLRP3 inflammasome/GSDMD pathway to protect cardiomyocytes from pyroptosis, thereby increasing cell survival and ameliorating cardiac dysfunction. Considering its protective effect on cardiomyocytes, rh PLTP could be a promising agent for treating SICD.
